# Nanoblinker: Brownian Motion Powered Bio-Nanomachine for FRET Detection of Phagocytic Phase of Apoptosis

**DOI:** 10.1371/journal.pone.0108734

**Published:** 2014-09-30

**Authors:** Candace L. Minchew, Vladimir V. Didenko

**Affiliations:** 1 Baylor College of Medicine, Houston, Texas, United States of America; 2 Michael E. DeBakey Veterans Affairs Medical Center, Houston, Texas, United States of America; University of California, San Diego, United States of America

## Abstract

We describe a new type of bio-nanomachine which runs on thermal noise. The machine is solely powered by the random motion of water molecules in its environment and does not ever require re-fuelling. The construct, which is made of DNA and vaccinia virus topoisomerase protein, can detect DNA damage by employing fluorescence. It uses Brownian motion as a cyclic motor to continually separate and bring together two types of fluorescent hairpins participating in FRET. This bio-molecular oscillator is a fast and specific sensor of 5′OH double-strand DNA breaks present in phagocytic phase of apoptosis. The detection takes 30 s in solution and 3 min in cell suspensions. The phagocytic phase is critical for the effective execution of apoptosis as it ensures complete degradation of the dying cells’ DNA, preventing release of pathological, viral and tumor DNA and self-immunization. The construct can be used as a smart FRET probe in studies of cell death and phagocytosis.

## Introduction

Previously described nanomachines rely on a continuous re-supply of energy carriers, such as ATP, DNA or UV light [1], [2]. A nanodevice driven by Brownian motion would harvest energy from the environment and would not require re-fuelling. In this report we present such a new type of bio-nanomachine which runs on thermal noise. It is powered by the random motion of surrounding water molecules. The biomolecular construct, which we called nanoblinker, emits alternating pulses of green and red light by utilizing FRET. It uses a viral topoisomerase as an internal clock to repeatedly cut and restore a phosphodiester bond in a staple-shaped oligonucleotide. This permits employing Brownian motion as a cyclic motor to drive continuous separation and re-association of two types of fluorescent hairpins. The resulting molecular device performs its function by going through rounds of detachment and recombination of its parts.

The construct is an ultra-fast and specific probe for 5′OH double-stranded DNA breaks which mark the phagocytic phase of apoptosis [3], [4]. We demonstrate its utility by detecting this process in cultured cells in 3 minutes.

**Figure 1:**
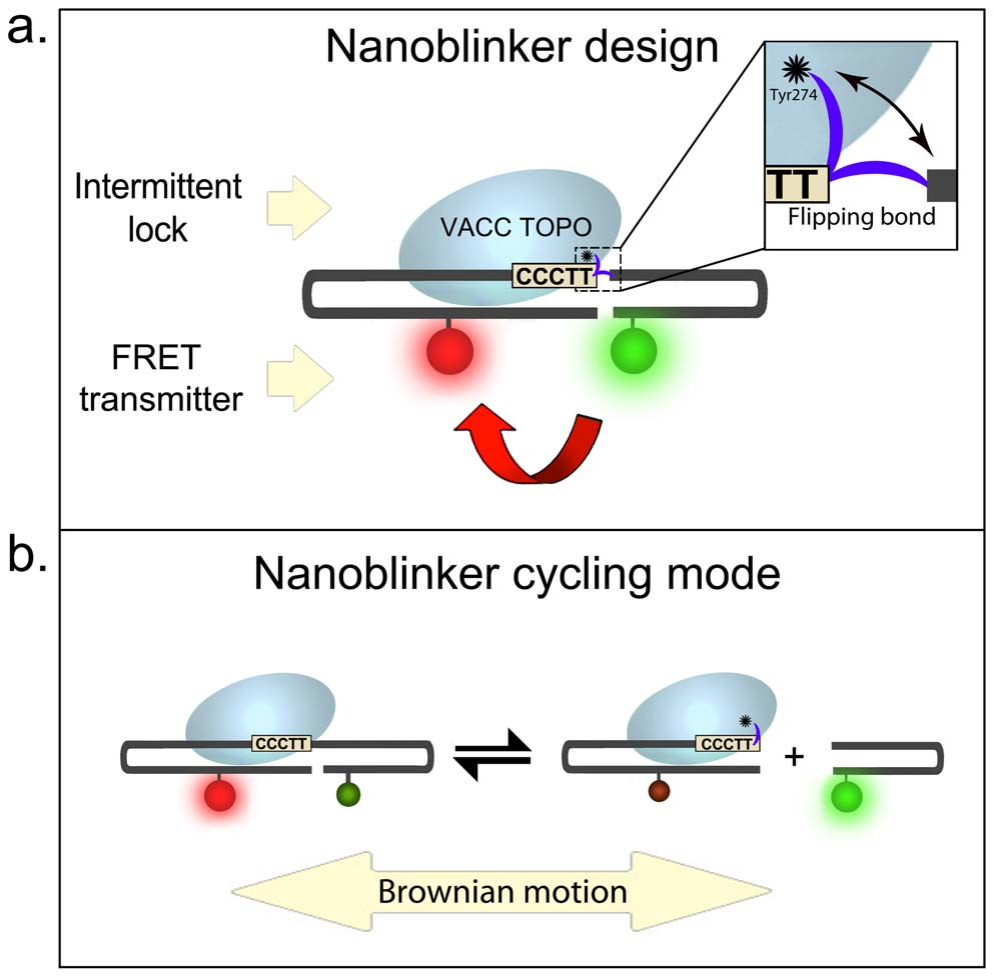
Components of nanoblinker and its mode of operation. **a.** Parts of nanoblinker; **b**. FRET “on” and “off” cycles powered by Brownian motion.

Importantly, the nanoblinker is not just a DNA damage sensor, it is also a FRET oscillator solely powered by the random motion of water molecules in its environment. Its design scheme presented from the nanomachine standpoint is shown in **Figure 1**.

**Figure 1** shows that, unlike many previously reported nanoscale devices, our construct does not emulate mechanical macromachines. The device does not maintain a permanent frame. Its parts form pairs which continuously separate and reattach back. Moreover, the pair members can freely exchange between individual pairs as opposed to being bound in permanent duets.

This mode of action has not been used before in artificial nanodevices; however it is widely employed by enzymatic molecular machines in living cells. The prime example of this work mode is provided by G proteins. The heterotrimeric G proteins are ubiquitous molecular regulators, which couple activation of cell surface receptors to intracellular responses controlling transcription, motility, contractility, and secretion [5]. These natural molecular machines function by going through cycles of complete separation and re-association of their Gα and Gβγ subunits. In their cyclic recombinations they are not constrained to permanent sets [6], [7].

**Figure 1** shows that there are three functional components in a nanoblinker: 1. an intermittent enzymatic lock, 2. a FRET-based transmitter, and 3. the Brownian motion used as a motor.

The intermittent lock function is performed by vaccinia topoisomerase I (VACC TOPO), a virus-encoded protein [8]–[10]. VACC TOPO repetitively breaks and restores the phosphodiester bond at the end CCCTT3′ sequence. The bond is the only link between two opposing hairpins. Depending on its condition, the hairpins are intermittently locked together or permitted to separate.

VACC TOPO is a IB type topoisomerase, a naturally occurring molecular machine which unwinds DNA in cells infected by vaccinia virus [11]. It is ATP-independent and instead relies on the torque of coiled DNA strands to perform unwinding, while the enzyme repetitively breaks and rejoins one strand of the DNA duplex [12]. Our design does not utilize this torque as a source of energy and exploits only the ability of VACC TOPO to cyclically cut and restore a phosphodiester bond. In nature this leads to rapid reversible cleavage and re-joining of DNA stands during DNA unwinding. In our construct this intermittent lock-release plays the role of internal timer, analogous to the quartz crystal in computer clocks.

Another part of the nanoblinker is a staple-shaped oligonucleotide (Core Oligo). It works as FRET-based signal transmitter. Its purpose is to indicate target detection. The Core Oligo is a self-complimentary 38-mer dual-hairpin containing CCCTT3′ sequence recognized by VACC TOPO (**Fig. 1a**). The oligo also carries a FAM-TAMRA donor-acceptor pair. Spontaneous folding of the oligomer brings donor and acceptor fluorophores inside their Förster radius (**R_0_**) range, activating FRET. The estimated distance between FAM and TAMRA in the folded Core Oligo is 23.8 Å which is within the 55 Å **R_0_** for this pair [13]. Therefore in the folded oligo TAMRA (red) is radiative, whereas the FAM fluorescence (green) is suppressed.

Nanoblinkers self-assemble and self-activate when VACC TOPO is combined with the Core Oligo. The enzyme specifically attaches to the CCCTT3′ site and cleaves the phosphodiester bond in CCCTT ↓3′ by exploiting Tyr274 nucleophile. It results in the covalent attachment of VACC TOPO to the 3′ end of the oligo and the expulsion of a 5′ OH -terminated DNA. This breaks the Core Oligo into two separate hairpins, one carrying the enzyme and another enzyme-free (**Fig. 1b**). If not immediately separated, the two hairpins would ligate back and would then again re-cleave.

The recurring cleavage-religation permits separation and random re-association of the hairpins through continuous collisional impacts with surrounding water molecules. This bombardment provides the source of movement, serving as a motor for the hairpins, which perpetually sever and restore their link (**Fig. 1b**).

The cleavage and religation steps are reversible [14], [17] and require no outside biochemical energy carriers, such as ATP, for continuous repetition [15], [16]. In the steady-state kinetics studies of VACC TOPO, the enzyme was shown to proceed through ≥22 such cleavage-religation cycles during 300 seconds of observation [17].

Such periodic changes, taking place in the mechanical plane, manifest in alternating fluorescence outputs from individual donor- and acceptor-bearing hairpins. During this process donor and acceptor fluoresce in counterphase as each fluorophore goes thorough successive cycles of fluorescence and dimming. The lengths of these radiative and non-radiative phases for donor or acceptor are controlled by the condition of the Core Oligo (ligated or cleaved).

As expected, on the basis of 1 kcal/mol free energy difference between tyrosine and ribose phosphodiesters [18], the cleavage-religation equilibrium for VACC TOPO favors the ligated state by an order of magnitude [17]. Therefore most time during each cycle the nanoblinkers are uncut and form a single complex. They go through brief periods of separation before recombining back into the covalently-linked constructs. For this reason the FRET acceptor is radiative most of the time, whereas the donor is only transiently fluorescent.

This “blinking” system is a sensitive detector of specific DNA damage because the length of the donor fluorescence phase, when the hairpins are separated, radically increases in the presence of additional 5′OH DNA breaks. A blunt-ended 5′ OH DNA break represents the selective alternative target for the acceptor-carrying hairpin with bound VACC TOPO, which can ligate to it instead of the donor-labeled hairpin. This stops FRET, extinguishes fluorescence of the acceptor and stabilizes the donor in the radiative phase. As a result, the color of emitted fluorescence changes which signals detection.

## Results

### Demonstration of all stages of the “idling” nanoblinker system and specific detection of the added 5′OH DNA acceptors

**Figure 2:**
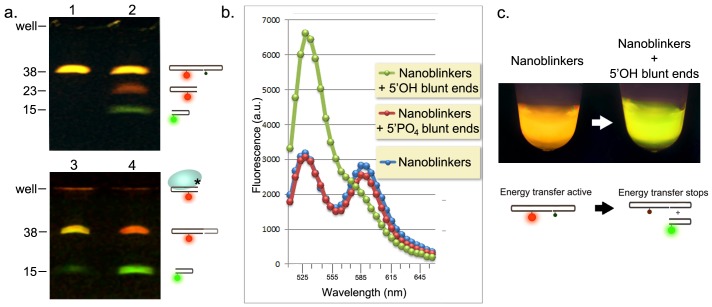
Detection of blunt-ended 5′OH DNA in model systems using nanoblinkers. **a. PAGE separation of working parts of nanoblinker:** *Lane 1*. 38-mer Core Oligo;*Lane 2*. Cycling nanoblinkers assembled by combining Core Oligo with VACC TOPO: equilibrium between 15-mer +23-mer (green and red bands) and 38-mer Core Oligo (yellow fluorescence). Solution was treated with proteinase K to strip VACC TOPO (10 µg/µL, 15 min, 37°C). *Lane 3.* Same as Lane 2, but without treatment by proteinase K. VACC TOPO–23-mer complex did not enter the 20% acrylamide gel and was retained in the well (red fluorescence). *Lane 4.* Detection of 5′ OH blunt ended DNA ends. Unlabeled 21-mer Test Oligo was added to the cycling nanoblinkers. Detection reaction created new chimeric 44-mer (red fluorescence). Compare with dual labeled Core Oligo in Lane 3 (yellow fluorescence). Note greater intensity of the green band due to the release of the 15-mer hairpin as a result of the detection reaction. **b.** **FRET-based detection of 5′OH DNA ends**. Emission spectra of cycling nanoblinkers before (*blue curve*) and after (*green curve*) addition of 5′OH DNA ends - Test Oligo (to final concentration 20 pmol/µL). Addition of same amount of 5′PO_4_ DNA ends (Test Oligo PO_4_ ) did not change fluorescence (*red curve*). λ_Excitation_ - 488 nm. For Probes and nanoblinker Core Oligo sequences see Materials. **c. Image of the tubes with nanoblinkers taken through the objective of a fluorescence microscope before (left tube), and after (right tube) addition of 5′OH DNA ends.** λ_Excitation_ - 490 nm.

The nanoblinkers were assembled as described in Methods and tested in the model system with predetermined concentrations of specific DNA ends. All components of the working nanoblinker system, as presented in the schematic in **Figure 1**, were observed after separation by polyacrylamide gel electrophoresis (PAGE) (**Fig. 2a**). The discrete bands in the gel demonstrate: the uncleaved Core Oligo prior to the reaction (yellow 38-mer in lane 1), both separated hairpins (green 15-mer and red 23-mer bands in lane 2), and the new chimeric 44-mer DNA duplex (red band in lane 4) which formed after the attachment of the 23-mer VACC TOPO-carrying hairpin to the unlabeled Test Oligo.

The observation of all stages of the “idling” nanoblinker system and the “detection” of the added 5′OH DNA acceptor, i.e. the formation of the chimeric TAMRA-labeled 44-mer, indicated that the construct went through the working cycle shown in **Figure 1**.

**Figure 2b** presents spectrofluorimetric tests of the nanoblinker system detecting its target DNA breaks and its reaction to the addition of non-target DNA ends.

The emission spectrum of the “idling” nanoblinkers (i.e. no added DNA ends - *blue curve*) shows the peaks of similar heights at 580 nm and 525 nm indicating radiative TAMRA (acceptor) and suppressed fluorescence of FAM (donor). This confirmed the ongoing FRET and verified that in the idling stage nanoblinkers were predominantly in the ligated form.

After the addition of blunt ended 5′PO_4_ DNA to 20 pmol/µL (*red curve*) the spectrum did not change indicating the complete absence of reaction with this non-target DNA. Moreover, the very presence of a substantial amount of non-reacting DNA ends in the solution had no effect on fluorescence.

In sharp contrast, the addition of target blunt ended 5′OH DNA to 20 pmol/µL (*green curve*) resulted in a shift of the emission maximum from 580 nm to 525 nm within 30 seconds. This was accompanied by an increase of the FRET ratio (E_D/A_ = E_D 525 nm_/E_A 580 nm_ [19], [20]) from 1.12 to 3.24, indicating the unmasking of donor and suppression of acceptor fluorescence. This high speed of detection was supported by the intrinsically fast ligation activity of VACC TOPO. In the earlier kinetic analysis of DNA strand cleavage and ligation reactions this enzyme demonstrated ligation of 85% of oligoprobes within a 15 sec interval [17].

**Figure 2c** shows images of the tubes with nanoblinkers before and 30 seconds after the addition of 5′OH DNA breaks. The images were taken through the objective of a fluorescence microscope. They illustrate the extent of the fluorescence color shift due to FRET disruption produced in detection of specific DNA breaks.

Overall the data presented in **Figure 2** confirmed that the construct could very rapidly and specifically detect 5′OH blunt-ended DNA breaks, discriminating them from the structurally similar 5′PO_4_ blunt-ended DNA. To perform this task the sensor had to cleave itself at least once and then selectively re-ligate to the breaks.

### Demonstration of continuous cleavage-religation of nanoblinkers and specific detection of DNA added 30 seconds and 15 minutes after the nanoblinker system assembly

The experiments described above verified that nanoblinkers underwent cleavage and religation after their initial self-assembly. However, the results did not answer the question of whether these events occurred only once or were continuously repeated.

This was important to establish because the observed FRET disruption during the detection step could have resulted from just few cleavage-religation cycles after which the system “froze” due to the exhaustion of either the cleavage or the ligation activity of VACC TOPO. In such a case the specific DNA breaks would still be detected and signaled by the permanent disruption of FRET caused by either the inability of separated hairpins to religate back or their failure to disattach from the acceptor DNA after the ligation. In both of these scenarios the nanoblinker system would be unable to restore the Core Oligo and reestablish FRET shortly after the reaction initiation.

Such a short-lived oscillator would still be an ultra-fast and specific sensor of DNA breaks, but it would rapidly deactivate, and would have to be used right after its preparation. One consequence of this would be the loss of the ability to respond to new breaks if they are added a short time later, when the cleavage-ligation activity of the topoisomerase is exhausted. Thus, additional experiments were performed to demonstrate the perpetual cycling between cleaved and ligated phases during extended periods of time.

The experiments tested the ability of the cycling nanoblinker system to respond to new breaks when they were added 15 minutes post assembly. In these experiments we used an unlabeled variant of the Core Oligo (Core Oligo-2) and two 5′OH blunt-ended DNA acceptors with identical sequences but labeled with two different fluorophores (FAM Test Oligo and TAMRA Test Oligo). The contrasting green and red fluorophores were used to separate the chimeric 38-mers made from the early (30 s) and the late (15 min) added DNA.

The responsive system would “detect” the newly added 5′OH DNA breaks by synthesizing the alternatively-colored set of chimeric 38-mers from the fluorescent 15-mers and the non-fluorescent 23-mer parts of the Core Oligo-2. Consequently the first set of fluorescent acceptors was added to nanoblinkers immediately (30 s) after their preparation and the alternatively labeled set of acceptors was added to the same solution 15 minutes later. The reactions were stopped at 30 minutes (see **Methods**).

**Figure 3:**
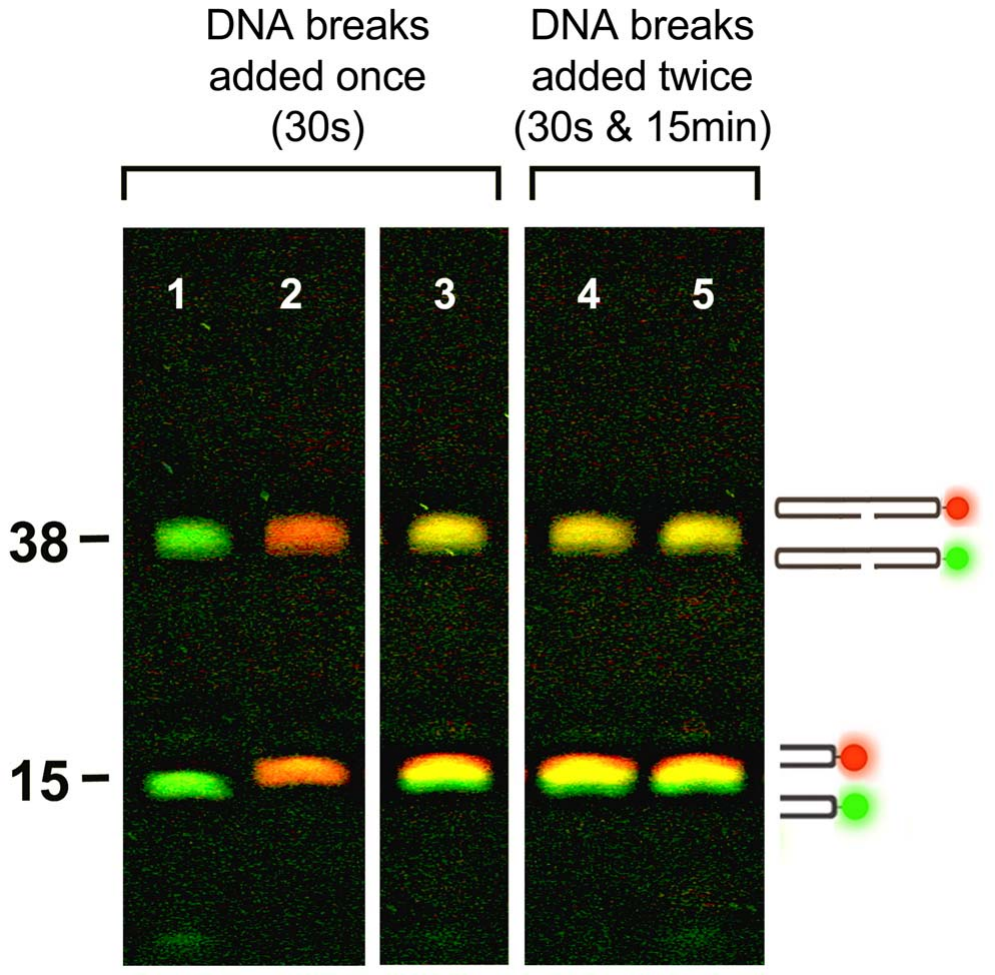
Detection of DNA breaks added to the nanoblinker system 30 s and 15 min after the initiation of the cycling reaction. **PAGE separation of fluorescent “detection” products of the cycling nanoblinker system. Nonfluorescent cycling nanoblinker system (38-mer-VACC TOPO complex ↔ 15-mer +23-mer-VACC TOPO complex) created by mixing 100 pmoles Core Oligo-2+100 pmoles VACC TOPO. Green and red fluorescent acceptors added at 30 s and 15 min post assembly:** *Lane 1*. 100 pmoles FAM-labeled 15-mer FAM Test Oligo (green) added to the cycling system at 30 s; *Lane 2*. 100 pmoles TAMRA-labeled 15-mer TAMRA Test Oligo (red) added to the cycling system at 30 s; *Lane 3*. 100 pmoles FAM-labeled 15-mer FAM Test Oligo (green) +100 pmoles TAMRA-labeled 15-mer TAMRA Test Oligo (red) added to the cycling system at 30 s; *Lane 4*. 100 pmoles FAM-labeled 15-mer FAM Test Oligo (green) added to the cycling system at 30 s +100 pmoles TAMRA-labeled 15-mer TAMRA Test Oligo (red) added to the cycling system at 15 min; *Lane 5*. 100 pmoles TAMRA-labeled 15-mer TAMRA Test Oligo (red) added to the cycling system at 30 s +100 pmoles FAM-labeled 15-mer FAM Test Oligo (green) added to the cycling system at 15 min; The reactions were stopped at 30 minutes. Solutions were treated with proteinase K to strip VACC TOPO (10 µg/µL, 15 min, 37°C). Note the 38-mer bands representing the “detection” products. The yellow color in *Lane 3* is produced due to superimposition of fluorescence from red and green 15-mers added at the 30 s time-point and “detected” by ligating to 23-mer-VACC TOPO hairpins. Compare with equally intense yellow 38-mer bands in *Lanes 4, 5* produced by “detection” of red and green 15-mers mix added separately at 30 s and 15 min time-points.

The results of these experiments are presented in **Figure 3** **.** They demonstrate that after 15 minutes of cycling, the system was still highly responsive to the addition of new breaks. It actively synthesized the chimeric fluorescent 38-mer hairpins with no observable difference between the early and late series (**Fig. 3** *Lane 3* ***vs*** *Lanes 4, 5)*.

Earlier studies of VACC TOPO demonstrated the completion of the ligation of 85% of oligoprobes within a 15 sec interval [17]. Therefore, for the “detection” to proceed beyond that time period, the 23-mer-VACC TOPO intermediates should be constantly re-cleaved from the ligated constructs. Moreover, the detection of the newly added DNA ends by such an actively re-ligating system would have occurred only if after their re-cleavage the parts were continuously and vigorously separated and recombined by collisional impacts. Brownian motion, the source of these continuous impacts, provides a cyclic motor function for the oscillating system.

Overall the results confirmed ongoing cleavage-religation in the nanoblinker system and its ability to detect new 5′OH blunt-ended DNA ends 15 minutes post assembly.

Importantly, the steady-state kinetics studies of VACC TOPO, done earlier, have also reported continuous cleavage-religation in the VACC TOPO-oligonucleotide system during 300 seconds of observation. During that time the enzyme was shown to carry on ≥22 cleavage-religation cycles [17].

### Demonstration of the intermittent lock function of VACC TOPO in detection reactions

The cleavage-religation equilibrium favors the ligated state by an order of magnitude [17], therefore most nanoblinker dual hairpins are uncut (FRET “turned on” – **Fig. 2c** **.** *red fluorescence*). However the detection can only occur when the dual hairpins are cut and the active ligatable 23-mer-VACC TOPO complexes are available. Yet after each cleavage, 23-mer-VACC TOPO rapidly disappear by ligating back due to the 10-fold prevalence of ligation over cleavage. Therefore, to maintain the ability of nanoblinkers to detect, the cuts must be constantly repeated. Moreover, less frequent cutting would slow down the “ticking” of the system, lengthening the detection time, whereas increased cleavage would have the opposite effect. For that reason, the lock-release function of VACC TOPO is made evident in detection reactions when changes in the cleavage activity of the enzyme are closely followed by changes in the time required for detection of added DNA breaks.

In the next series of experiments we investigated the influence of the cleavage activity of VACC TOPO in nanoblinkers on the time frame of DNA damage detection.

**Figure 4:**
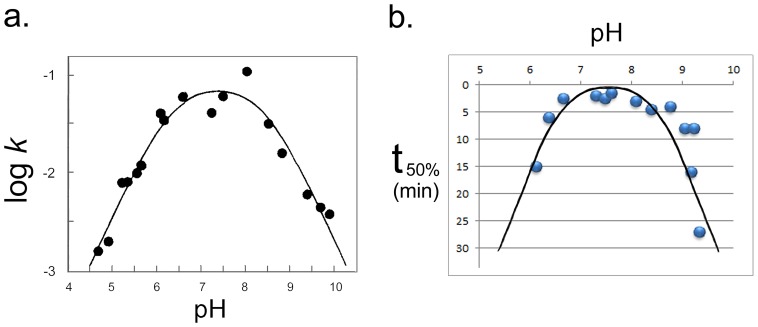
Changes in VACC TOPO cleavage activity are followed by changes in t_50%_ detection time. **a. pH-induced changes in the VACC TOPO rate constant for strand cleavage.** The first-order rate constants for strand cleavage in the pH range 4.6–9.8 obtained using single-turnover conditions **(**from 21 with permission). **b. pH-induced changes in the time of nanoblinker-based detection of 5′OH DNA breaks (t_50%_).** Detection time **t_50%_** - time interval in minutes required to reach a 50% increase in the starting FRET ratio for each individual pH value.

It is well-established that the cleavage activity of VACC TOPO is selectively and specifically altered by pH changes [21]. The VACC TOPO rate constant for strand cleavage ***(k_cl_)*** is highly dependent on pH over the range 4.5–9.5, showing a bell-shaped profile with apparent pK_a_ values of 6.3±0.2 and 8.4±0.2 (**Fig. 4a**). In contrast, the VACC TOPO rate constant for strand religation ***(k_r_)*** is independent of pH over that range. Importantly, the pH dependence of ***k_cl_*** is solely due to the internal conformational changes in the enzyme, not in the oligonucleotide test system such as ours [21]. Therefore, in the experiments we monitored the nanoblinker-based detection of 5′OH DNA breaks in the pH range 6–9. The time intervals required to reach a 50% increase in the starting FRET ratio (**t_50%_**) were determined for each individual pH value. These are presented in **Figure 4b**.

The figure shows that **t_50%_** displays a bell-shaped profile which closely follows the pH-induced changes in cleavage activity (**Fig. 4a**). As neither the ligation activity of VACC TOPO, nor the attachment of the enzyme to oligos, were affected by pH changes in the range 6–9 [21], this indicates that stronger cleavage in nanoblinkers was accompanied by faster detection and weaker cleavage resulted in delayed detection.

### Cell culture application of nanoblinkers for express detection of phagocytosis. Discrimination between phagocytosis of apoptotic and necrotic cells

In the previous experiments we established that nanoblinkers reacted exclusively with 5′ OH blunt ended DNA breaks with no reactivity towards 5′PO_4_ blunt ended DNA (**Fig. 2**). We used this selective property in the cell culture application of this system.

Apoptosis and phagocytic digestion of DNA are the only two processes in cells which specifically generate blunt ended DNA breaks. Non-programmed necrotic cell death or random DNA damage do not produce this type of DNA cleavage [22]–[24]. Although both apoptosis and phagocytosis produce blunt ended DNA breaks, they differ in a principal way because their end-group patterns are inverted. Apoptotic DNA breaks, produced by executioner nucleases, have 3′OH/5′PO_4_ configuration, whereas DNA breaks generated in phagocytic digestion by DNase II have the inverted 3′PO_4_/5′OH configuration [3], [25]. These are very stable characteristics which do not change until complete dissolution of the nuclei [26].

As we demonstrated above, nanoblinkers could selectively detect the second, but not the first type of DNA cleavage. Therefore we tested them in cell cultures as specific sensors of phagocytic engulfment.

In experiments we assessed phagocytosis of apoptotic and necrotic U87 cells by cultured phagocytic J774A.1 macrophage cells. Apoptosis and necrosis were verified both morphologically and biochemically (see Table S1 and *Cell cultures, apoptosis, necrosis, and phagocytosis induction)*. Control experiments showed that nanoblinkers added to apoptotic or necrotic cells *per se* did not produce signal (see Tables S2–S3). This confirmed that the target 5′OH DNA breaks were not generated in these cells before phagocytic engulfment.

**Figure 5:**
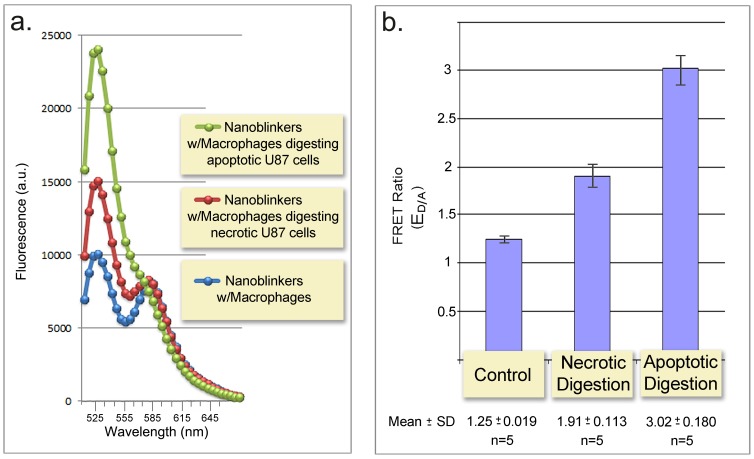
FRET-based detection of phagocytosis of apoptotic and necrotic cells. **a.** Emission spectra of nanoblinkers with macrophages before phagocytosis initiation (*blue curve*), and 3 min after the addition of macrophages digesting apoptotic (*green curve*) or necrotic cells (*red curve*). λ_Excitation_ - 488 nm. (Full spectra and graphical representations of all experiments and additional controls see Tables S2–S3). **b.** FRET ratios (E_D/A_) of nanoblinkers detecting phagocytosis of apoptotic and necrotic cells by macrophages. Control - macrophages before phagocytosis initiation by addition of apoptotic or necrotic cells. When tested separately before phagocytosis all cell types (macrophages, apoptotic and necrotic U87 cells) produced indistinguishable low intensity signals (see Tables S2–S3). The spectrofluorimetric assessment presented as Mean ± SD for five independent experiments was performed 3 min post addition of nanoblinkers (see *Spectrofluorimetric assessment of phagocytosis using nanoblinkers* (E_D/A_ = E_D 525 nm_/E_A 580 nm_ ); λ_Excitation_ - 488 nm.

The results of the phagocytosis assessment are presented in **Figure 5** and demonstrate that nanoblinkers detected phagocytic digestion of both apoptotic and necrotic cells. The detection time was 3 minutes. Moreover, the detection clearly distinguished apoptotic cell phagocytosis from that of necrotic cells (**Fig. 5**).

The likely molecular basis of such discriminative detection is in the different intensity of phagocytic clearance of apoptotic vs. necrotic cells. In subsequent experiments we demonstrated this in application to the cell culture model of phagocytosis which we employed in this study.

This time we used nanoblinkers as *in situ* probes to fluorescently tag phagolysosomes in macrophages digesting apoptotic and necrotic cells. Nanoblinkers were applied to the cells grown on glass chamber slides. The slides were washed and analyzed under a fluorescence microscope (see **Methods**). In this case the un-attached parts of nanoblinkers were washed away and the signal was created without FRET, but by direct fluorescence of the fluorophore of the 23-mer hairpin (green in case of this preparation of nanoblinkers).

**Figure 6:**
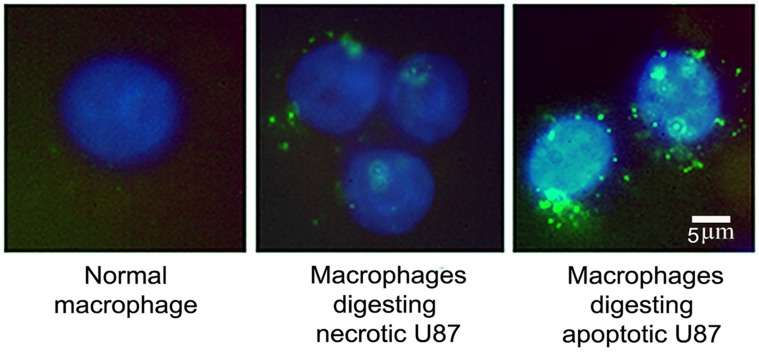
Fluorescence microscopy of macrophages digesting apoptotic and necrotic cells. Phagolysosomes in the cytosol of macrophages are the organelles which process apoptotic and necrotic cells and are labeled by the nanoblinker construct (green). Nuclei of macrophages are visualized by fluorescence stain DAPI (blue). Note more active phagocytosis in case of apoptotic cells. λ_Excitation_ - 488 nm. FITC excitation D490/40, emission 520/10; DAPI.

Fluorescence microscopy of the labeled cells revealed significantly higher numbers of phagolysosomes digesting material from apoptotic rather than from necrotic cells (**Fig. 6**).

We conclude that nanoblinkers can be used as rapid and specific sensors capable of detecting phagocytosis in cell cultures, distinguishing between apoptotic and necrotic cell engulfment.

## Discussion

To better understand these results from the cell biology perspective, it is important to note that the nature of cell death (apoptotic or necrotic) is an essential characteristic, which affects phagocytic intensity [3], [27]. The engulfment of necrotic cells is relatively slow, whereas apoptotic cell corpses are rapidly removed not only by professional phagocytes – macrophages, but also by neighboring cells [3], [22]. This is because, unlike necrotic, apoptotic cells express “find-me”, “bind-me”, “eat me”, “clear-me”, etc. signals stimulating phagocytosis [27]. The importance of phagocytic clearance of apoptotic cells prompted its recent inclusion in the apoptotic process as its final phagocytic waste management phase [3], [4]. Therefore it became essential to have a broad-spectrum probe which could detect a general marker of engulfment and label all the various cell types participating in phagocytic clearance in live cell suspensions. Such suspensions, ranging from cultured cells to clinical biopsy specimens and blood test samples, are widely used in cell biology and biomedical research. From this perspective the nanoblinker can be utilized as a new and advantageous sensor useful in apoptosis studies.

In its application as a FRET probe the nanoblinker has a special advantage in homogenous systems. In such systems (solutions or cellular preparations) the unreacted probe cannot be “washed out” like it is in tissue sections or in fixed cells. Therefore it is essential for the probe to send a fluorescent signal only when its targets are present.

Another peculiar feature of this system is in its ability to respond with color change when its target’s concentration changes (see **Fig. 3**). This is because the VACC TOPO hairpin continually fluctuates between attaching to the target and to the second fluorescent hairpin. It opens the possibility of using nanoblinkers to monitor changes in the environment and will be investigated in our future research.

All of these characterize the nanoblinker as a “smart” probe and principally distinguish it from *in situ* probes for detection of the apoptosis phases in tissue sections developed earlier [24], [28].

By being both a nanodevice and a biological probe the nanoblinker bridges the fields of bio-nanotechnology and cell biology. Analysis of its operation principles can be instrumental for the development of future molecular-scale appliances.

The operation of nanoblinkers is driven by random molecular collisions with surrounding water molecules. To harness these extremely frequent and disorderly impacts the nanoblinker continually cuts and re-ligates itself. These regular changes are controlled by the intermittent enzymatic lock - VACC TOPO. The only transformation inflicted by the enzyme is a periodic change in the condition of the phosphoester bond at the 3′ DNA end. The bond constantly fluctuates between Tyr274 in the enzyme and the opposing 5′ OH DNA end. As a result, the parts of the nanoblinker are intermittently either permitted or restricted to move. When the bond is at Tyr274, the two parts of the nanoblinker are completely separated. Without Brownian motion they would stay close and would re-ligate, however due to bombardment by water molecules, they are prone to move apart. This allows nanoblinkers to use Brownian motion the same way a sailboat would use wind. Eventually the construct will re-form when the Tyr274-bearing hairpin is brought close to any 5′ OH-ended hairpin in the surrounding solution (either the same it separated from, or any other). Similar subunit separation and exchange are common in biological machines [6], [7].

Consequently the described DNA-protein system makes use of chaotic forces in the environment. However, unlike molecular ratchets of directional enzymatic motors [29], this mechanism does not provide a uni-directional bias. It only ensures constant repetition of the same sustained cycle of transformations.

The nanoblinker cannot move directionally and does not need it because its random passage through the whole volume of the solution is a more efficient search strategy for its targets. In this regard Brownian motion is the most efficient propellant, as it supplies ∼10^−8^ W per molecule through the random environmental buffeting at room temperature, compared to the maximum power output of 10^−16^ W of a typical motor protein fueled by ATP [30].

Thus in our setup, VACC TOPO works as a recurrent lock-release mechanism, whereas all motor function is outsourced to Brownian motion, making the environment an essential working part of the machine. This sharing of the functional load with the environment permits a relatively simple construct to exhibit robotic-type behavior and to perform a complex set of tasks including target search, detection, and signaling.

## Methods

### Materials

Core Oligo – a 38-mer dual-labeled with FAM and TAMRA:

5′-AAGGG**T(TAMRA)**CCTGCTGCAGGACCCTTAACGCATTATGCG**T(FAM)**T-3′.

Core Oligo 2 - a 38-mer unlabeled:

5′-AAGGGTCCTGCTGCAGGACCCTTAACGCATTATGCGTT-3′.

Core Oligo 3 - a 38-mer labeled with FAM:

5′-AAGGGACCTGC**T(FAM)**GCAGGTCCCTTAACGCATTATGCGTT-3′.

Test Oligo – a 21-mer hairpin used to emulate the blunt-ended 5′ OH DNase II breaks in solution tests: 5′-GCGCTAGACCTGGTCTAGCGC-3′.

Test Oligo PO_4_– a 21-mer hairpin used to emulate non-target blunt-ended 5′ PO_4_ breaks in solution tests: 5′ -**(PO_4_)**GCGCTAGACCTGGTCTAGCGC-3′.

FAM Test Oligo – a 15-mer hairpin used to emulate the blunt-ended DNase II breaks in solution tests labeled with FAM: 5′-AACGCAT**T(FAM)**ATGCGTT-3′.

TAMRA Test Oligo – a 15-mer hairpin used to emulate the blunt-ended DNase II breaks in solution tests labeled with TAMRA: 5′-AACGCAT**T(TAMRA)**ATGCGTT-3′.

All oligonucleotides were synthesized and PAGE purified by IDT, Coralville, IA. On receipt they were diluted with bidistilled water to 100 pmol/µL concentration [Stock is 100 pmol/µL = 1.13 µg/µL] and stored at –20°C. Vaccinia DNA topoisomerase l was expressed in *E. coli* and purified as described [31].

### Cell cultures, apoptosis, necrosis, and phagocytosis induction

Phagocytic J774A.1 cells were purchased from ATCC (TIB-67). U87 cells were from ATCC (HTB-14). Apoptosis in U87 cells was induced by incubating at 42°C for 30 min. Cells were then returned to 37°C for 18 hours. Necrosis was induced by incubating cells at 65°C for 10 min. Cells were then returned to 37°C for 18 hours. Apoptosis and necrosis were verified by using the APO HTS 3/7 Caspase Detection kit (Cell Technology, Inc) and morphologically by fluorescence microscopy using DAPI staining (see Table S1). Phagocytosis was induced by combining treated U87 cultures (200 cells/well) with cultured J774A.1 macrophages (20 cells/well) for 18 hours at 37°C, 5% CO_2_.

### Spectrofluorimetric detection of 5′OH blunt ended DNA breaks in model system

The nanoblinkers were assembled in 100 mM Tris-HCl, pH 7.4 by combining 10 pmoles (2 pmol/µL final concentration) Core Oligo and 10 pmoles (2 pmol/µL final concentration) vaccinia topoisomerase I. 100 pmoles of Test Oligo or Test Oligo PO_4_ (20 pmol/µL final concentration) in 100 mM Tris-HCl, pH 7.4 was then added to the reaction mix. The plate was scanned 30 seconds later by using a Tecan Safire 2 scanning spectrofluorometer. Macro images of the tubes with the reaction were taken through the objective of a fluorescent microscope under 490 nm excitation light.

### PAGE tests of nanoblinker cycling at 30 s and 15 min post assembly

The cycling nonfluorescent nanoblinker system was assembled in 100 mM Tris-HCl, pH 7.4 by combining 100 pmoles (2 pmol/µL final concentration) Core Oligo-2 and 100 pmoles (2 pmol/µL final concentration) vaccinia topoisomerase I. This resulted in initiation of the cleavage-religation reaction: 38-mer-VACC TOPO ↔ 15-mer +23-mer-VACC TOPO (all unlabeled). 100 pmoles of FAM Test Oligo, TAMRA Test Oligo or a combination (FAM Test Oligo plus TAMRA Test Oligo) was then added to the reaction mix either in a single addition or as a 2 step addition (one at 30 s, then a second of the opposing color at 15 min). All reactions were stopped at 30 min and solutions were treated with proteinase K to strip VACC TOPO (10 µg/µL, 15 min, 37°C). Samples were run on a 20% acrylamide gel.

### Cleavage control experiments (pH-dependence)

The nanoblinkers were assembled in 100 mM Tris-HCl, at various pH ranging from 6–9.5, by combining 50 pmoles Core Oligo and 50 pmoles vaccinia topoisomerase I. 100 pmoles of Test Oligo was then added to the reaction mix. The plate was scanned every minute for 30 minutes using a Tecan Safire 2 scanning spectrofluorometer.

The time intervals (**t_50%_**) required to reach a 50% increase in the starting FRET ratio (E_D/A_ = E_D 525 nm_/E_A 580 nm_ [19], [20]) were determined for each individual pH value.

### Spectrofluorimetric assessment of phagocytosis using nanoblinkers

Following 18-hour incubation at 37°C, 5% CO_2,_ combined macrophage and U87 cell cultures (20 macrophages/200 U87 cells) were placed in a hypo-osmotic solution and vortexed to rupture cellular membranes_._ Solutions were combined with nanoblinkers (final concentration 500 fmol/µL in 100 mM Tris-HCl, pH 7.4). As controls, we used cells without 5′OH DNA breaks, such as normal macrophages, apoptotic U87 cells and necrotic U87 cells (Tables S2–S3). Donor fluorescence was measured 3 min post-addition at 488 nm excitation and 525 nm emission wavelengths using a Tecan Safire 2. All experiments were repeated 5 times.

### Visualization of phagocytosis using nanoblinkers in situ

Following 18-hour incubation at 37°C, 5% CO_2,_ combined macrophage and U87 cell cultures were washed and fixed on glass chamber slides for imaging. Nanoblinkers were assembled in 100 mM Tris-HCl, pH 7.4 by combining 100 pmoles of FAM labeled Core Oligo-3 and 100 pmoles vaccinia topoisomerase I. Slides were incubated for 15 minutes at room temperature (23°C) in a humidified chamber. Slides were washed in bidistilled water then covered with Vectashield with DAPI. Double-strand DNA breaks with 5′OH were labeled with green fluorescence.

### Microscopy and Imaging

Fluorescence Olympus IX-70 microscope with Chroma Technology band-pass filter set was used: FITC excitation D490/40, emission 520/10; DAPI excitation D360/40, emission 460/20. Images were recorded by an Olympus EVOLT digital SLR and a MicroMax digital video camera system (Princeton Instruments, Inc.).

### Statistical analysis

The Student’s t-test was used to determine statistical significance for the data from these experiments. Both the peak fluorescence of the donor at 525 nm and the FRET ratio (E_D/A_ = E_D 525 nm_/E_A 580 nm_) were analyzed. Statistical analysis was performed using NCSS software.

## Supporting Information

Table S1 **Verification of apoptosis in U87 cells via detection of apoptosis-specific caspase 3/7 activation.**
(DOC)

Table S2 **Verification that nanoblinker is not affected by cell suspensions which do not contain its target DNA breaks: normal non-phagocytizing macrophages, necrotic and apoptotic U87 cells.**
(DOC)

Table S3 **Full fluorescence spectra (a.u.) obtained in experiments testing nanoblinkers detecting phagocytizing macrophages.**
(DOC)
