# Supplementary material for: Nanoblinker: Brownian Motion Powered Bio-Nanomachine for FRET Detection of Phagocytic Phase of Apoptosis
Source: PLoS One. 2014 Sep 30;9(9):e108734. doi: 10.1371/journal.pone.0108734 (PMC4182547; doi:10.1371/journal.pone.0108734)
Supplement: Table S1 — Verification of apoptosis in U87 cells via detection of apoptosis-specific caspase 3/7 activation. (DOC) [file pone.0108734.s001.doc]

**Verification of apoptosis in U87 cells via detection of**

**apoptosis-specific caspase 3/7 activation**

**Table 1.**

|  | **Normal U87** | **Necrotic U87** | **Apoptotic U87** |
| --- | --- | --- | --- |
| Cell Series | U87 Control | U87 10' @ 65C | U87 30' @ 42C |
|  |  |  |  |
|  | Fluorescence at 525nm (a.u.) | Fluorescence at 525nm (a.u.) | Fluorescence at 525nm (a.u.) |
|  |  |  |  |
|  | 4771 | 9498 | 23345 |
|  | 9997 | 23156 | 17366 |
|  | 10770 | 8934 | 14007 |
|  | 10280 | 10238 | 14137 |
|  | 12686 |  |  |
|  | 10965 |  |  |
|  |  |  |  |
| SUM | 59469 | 51826 | 68855 |
|  |  |  |  |
| **MEAN** | **9911.5** | **12956.5** | **17213.75*** |
|  |  |  |  |
|  |  | p=0.3436 | p=0.0107 |
|  | SD=2687.47 | SD=6820.6 | SD=4372.83 |
|  | N=6 | N=4 | N=4 |
|  |  |  |  |
|  |  |  | ***** **Statistically significant** |

Apoptosis was verified by the homogenous fluorimetric assay for active caspase 3/7, using λ _excitation_ = 488 nm, λ _emission_ = 525 nm (Cell Technology, Inc.).

Apoptotic or necrotic morphology of U87 cells in different series was confirmed by fluorescence microscopy using DAPI staining.
