# Supplementary material for: Nanoblinker: Brownian Motion Powered Bio-Nanomachine for FRET Detection of Phagocytic Phase of Apoptosis
Source: PLoS One. 2014 Sep 30;9(9):e108734. doi: 10.1371/journal.pone.0108734 (PMC4182547; doi:10.1371/journal.pone.0108734)
Supplement: Table S2 — Verification that nanoblinker is not affected by cell suspensions which do not contain its target DNA breaks: normal non-phagocytizing macrophages, necrotic and apoptotic U87 cells. (DOC) [file pone.0108734.s002.doc]

**Verification that nanoblinker is not affected by cell suspensions which do not contain its target DNA breaks: normal non-phagocytizing macrophages, necrotic and apoptotic U87 cells.**

|  | **Macrophages Only** | **Necrotic U87 Only** | **Apoptotic U87 Only** | **Macrophages digesting necrotic U87** | **Macrophages digesting apoptotic U87** |
| --- | --- | --- | --- | --- | --- |
|  |  |  |  |  |  |
|  | Fluorescence at 525nm (a.u.) | Fluorescence at 525nm (a.u.) | Fluorescence at 525nm (a.u.) | Fluorescence at 525nm (a.u.) | Fluorescence at 525nm (a.u.) |
|  |  |  |  |  |  |
|  | 9768 | 8903 | 9303 | 16797 | 24022 |
|  | 10374 | 9094 | 9334 | 15943 | 26871 |
|  | 10640 | 9060 | 9260 | 14409 | 20926 |
|  | 10745 | 9150 | 9304 | 15032 | 21886 |
|  | 10032 | 7427 | 8241 | 14950 | 20897 |
|  |  |  |  |  |  |
| SUM | 51559 | 43634 | 45442 | 77131 | 114602 |
|  |  |  |  |  |  |
| **MEAN** | **10311.8** | **8726.8** | **9088.4** | **15426.2*** | **22920.4*** |
|  |  |  |  |  |  |
|  |  |  |  | p=0.0001 | p=0.0001 |
|  | SD=410.26 | SD=732.37 | SD=474.44 | SD=943.8 | SD=2547.67 |
|  | N=5 | N=5 | N=5 | N=5 | N=5 |
|  |  |  |  |  |  |
|  |  |  |  | ***** **Statistically significant** | ***** **Statistically significant** |

**Table 2.**

For each of the control series, cells were placed in a hypo-osmotic solution and vortexed to rupture cellular membranes and then were immediately added to the solution containing nanoblinkers at 500 fmol/μL in 100 mM Tris-HCl, pH 7.4. Concentrations of macrophages were 20 cells/well and U87 cells were 200 cells/well. Donor fluorescence was measured 3 min post-addition using λ _excitation_ = 488 nm, λ _emission_ = 525 nm.

Two right-side columns in the table represent phagocytizing macrophages which, unlike controls, contain target DNA breaks for the nanoblinker. These data were obtained simultaneously with the control experiments and are presented in detail in table 3 in Supplementary Materials. Only the values of fluorescence at 525 nm were extracted from that table and presented here for comparison with the non-phagocytic controls.
