# Supplementary material for: Nanoblinker: Brownian Motion Powered Bio-Nanomachine for FRET Detection of Phagocytic Phase of Apoptosis
Source: PLoS One. 2014 Sep 30;9(9):e108734. doi: 10.1371/journal.pone.0108734 (PMC4182547; doi:10.1371/journal.pone.0108734)
Supplement: Table S3 — Full fluorescence spectra (a.u.) obtained in experiments testing nanoblinkers detecting phagocytizing macrophages. (DOC) [file pone.0108734.s003.doc]

Full fluorescence spectra (a.u.) obtained in experiments testing nanoblinkers detecting phagocytizing macrophages.
Table 3.
Nanoblinker with Macrophages																																	
*	510nm	515nm	520nm	525nm	530nm	535nm	540nm	545nm	550nm	555nm	560nm	565nm	570nm	575nm	580nm	585nm	590nm	595nm	600nm	605nm	610nm	615nm	620nm	625nm	630nm	635nm	640nm	645nm	650nm	655nm	660nm	665nm	670nm	675nm	680nm	
A1	6733	8353	9575	9768	9126	8251	7216	6129	5441	5171	5362	5906	6639	7312	7656	7423	6944	6148	5133	4231	3414	2784	2331	1926	1643	1469	1258	1099	975	810	663	533	440	348	267	
A2	6860	8881	10160	10374	9925	8747	7583	6562	5903	5647	5800	6323	7100	7931	8302	8243	7690	6670	5666	4674	3803	3072	2558	2183	1865	1605	1443	1240	1012	883	710	552	469	373	323	
A3	6970	9166	10259	10640	9983	8947	7762	6758	5956	5759	5903	6497	7266	8149	8454	8353	7811	6878	5745	4675	3749	3117	2545	2191	1823	1628	1416	1255	1044	859	721	623	472	360	308	
A4	7006	9167	10337	10745	9948	8996	7802	6717	6018	5693	5908	6467	7317	8098	8532	8485	7895	6904	5880	4697	3821	3176	2595	2255	1846	1647	1453	1271	1068	866	735	618	470	378	306	
A5	6950	8758	9897	10032	9504	8499	7359	6310	5632	5423	5581	6087	6928	7533	8198	8010	7409	6454	5469	4511	3695	2951	2476	2157	1785	1571	1355	1181	978	840	690	558	446	363	306	
																																				
Nanoblinker with Macrophages digesting Necrotic U87																															
*	510nm	515nm	520nm	525nm	530nm	535nm	540nm	545nm	550nm	555nm	560nm	565nm	570nm	575nm	580nm	585nm	590nm	595nm	600nm	605nm	610nm	615nm	620nm	625nm	630nm	635nm	640nm	645nm	650nm	655nm	660nm	665nm	670nm	675nm	680nm	
B1	10822	14066	16272	16797	15776	14054	11936	10352	9059	8133	7662	7637	7913	8097	8072	7691	6971	6020	4984	4233	3361	2803	2276	1997	1674	1443	1283	1076	897	765	644	504	433	369	278	
B2	10392	13557	15492	15943	14809	13425	11428	9947	8506	7788	7415	7565	7943	8107	8222	7812	7067	6130	5239	4278	3449	2868	2347	1937	1700	1479	1267	1108	907	770	634	529	396	332	280	
B3	9489	12387	13999	14409	13514	11940	10374	8831	7761	7053	6936	7120	7596	7907	8009	7855	7087	6063	5275	4265	3466	2818	2365	1974	1720	1457	1290	1118	918	763	660	559	445	341	291	
B4	9942	12957	14750	15032	14129	12465	10852	9312	8158	7400	7217	7476	7837	8075	8266	7951	7325	6328	5424	4415	3565	2954	2376	2037	1745	1535	1341	1178	948	827	644	542	430	345	280	
B5	9909	12769	14593	14950	13883	12414	10646	9057	8093	7249	7012	7112	7502	7847	7875	7513	6885	5954	5105	4164	3359	2769	2304	1898	1647	1486	1244	1079	888	782	621	528	424	341	286	
																																				
Nanoblinker with Macrophages digesting Apoptotic U87																															
*	510nm	515nm	520nm	525nm	530nm	535nm	540nm	545nm	550nm	555nm	560nm	565nm	570nm	575nm	580nm	585nm	590nm	595nm	600nm	605nm	610nm	615nm	620nm	625nm	630nm	635nm	640nm	645nm	650nm	655nm	660nm	665nm	670nm	675nm	680nm	
C1	15806	20850	23796	24022	22582	20053	17108	14575	12604	10905	9970	9194	8631	8187	7505	6799	5911	5112	4266	3517	2915	2410	1990	1704	1425	1238	1050	877	730	613	484	416	342	271	233	
C2	17721	23237	26605	26871	25316	22402	19114	16301	13826	12181	10994	10196	9587	9229	8572	7678	6723	5754	4767	3893	3232	2708	2209	1869	1561	1379	1165	994	805	685	539	434	381	299	267	
C3	13726	17886	20480	20926	19437	17053	14736	12533	10770	9675	8870	8357	8021	7849	7516	6900	6075	5226	4500	3620	2944	2463	2049	1738	1449	1267	1064	927	769	642	538	417	353	321	238	
C4	14530	18794	21298	21886	20370	17889	15418	13045	11193	9988	9124	8735	8279	8082	7596	7025	6145	5364	4548	3702	3052	2477	2049	1776	1450	1281	1115	955	777	663	541	439	347	304	236	
C5	14867	18462	20678	20897	19459	17108	14743	12695	10735	9484	8603	8022	7569	7179	6711	6054	5383	4594	3869	3091	2601	2227	1755	1486	1303	1093	957	796	669	567	473	362	302	239	216	


Statistical analysis of 525 nm fluorescence peaks (a.u.).
				Mean		SD	n	P Value	
Group A: Nanoblinker with Macrophages	10311.8	±	410.26	5 		
Group B: Nanoblinker with Macrophages digesting necrotic U87	15426.2**	±	943.8	5 	p=0.0001	
Group C: Nanoblinker with Macrophages digesting apoptotic U87	22920.4**	±	2547.67	5 	p=0.0001	
** Statistically significant difference							


Graphical representations of all spectra for each series of experiments.

                                                                                                                                                                                                                   


The graphs show narrow distribution of values for each series. A slightly wider value distribution in the apoptosis series reflects higher variability in apoptosis induction in different cell samples, 
as compared to the more uniform necrosis reaction, when cells are evenly killed by the strong stimulus.  
The individual spectra (marked by circles in pink) showing median fluorescence values at 525 nm for each series, were chosen for Figure 3 representations in the paper.
